# Quenched Stochastic Optical Reconstruction Microscopy (qSTORM) with Graphene Oxide

**DOI:** 10.1038/s41598-018-35297-4

**Published:** 2018-11-16

**Authors:** Ruiheng Li, Pantelis Georgiades, Henry Cox, Sorasak Phanphak, Ian S. Roberts, Thomas A. Waigh, Jian R. Lu

**Affiliations:** 1 Biological Physics, School of Physics and Astronomy, University of Manchester, Oxford Rd., Manchester, M13 9PL UK; 2 Photon Science Institute, University of Manchester, Oxford Rd., Manchester, M13 9PL UK; 3 Division of Infection, Immunity and Respiratory Medicine, Michael Smith Building, Oxford Rd., M13 9PT Manchester, UK

## Abstract

Quenched Stochastic Optical Reconstruction Microscopy (qSTORM) was demonstrated with graphene oxide sheets, peptides and bacteria; a method of contrast enhancement with super-resolution fluorescence microscopy. Individual sheets of graphene oxide (GO) were imaged with a resolution of 16 nm using the quenching of fluorescence emission by GO via its large Resonant Energy Transfer (RET) efficiency. The method was then extended to image self-assembled peptide aggregates (resolution 19 nm) and live bacterial cells (resolution 55 nm, the capsular structure of *E. coli* from urinary tract infections) with extremely low backgrounds and high contrasts (between one and two orders of magnitude contrast factor improvements that depended on the thickness of the graphene oxide layer used). Graphene oxide films combined with STORM imaging thus provide an extremely convenient method to image samples with large backgrounds due to non-specifically bound fluorophores (either due to excess labelling or autofluorescent molecules), which is a common occurrence in studies of both biological cells and soft-condensed matter. The GO quenches the fluorescence across a thin layer at distances of less than 15 nm. Graphene oxide films coated with thin layers (≤15 nm) of polystyrene, polymethylmethacrylate and polylysine are shown to be effective in producing high contrast qSTORM images, providing a convenient modulation of sample/substrate interactions. The GO coatings can also provide an increased image resolution and a factor of 2.3 improvement was observed with the peptide fibres using a feature of interest metric,when there was a large non-specifically bound background.

## Introduction

Optical microscopy is a flourishing research field, in part due to the recent development of super-resolution fluorescence microscopy techniques, such as stochastic optical reconstruction microscopy (STORM)^1^, photoactivated localization microscopy (PALM)^2^, stimulated emission-depletion (STED) microscopy^3^ and structured illumination microscopy (SIM)^4^. The field provides many new possibilities for researchers to perform high quality non-invasive imaging experiments with a resolution approaching 20 nm. Contrast mechanisms in optical microscopy have also been subject to intensive research, e.g. the phase contrast methods of Zernicke^5^, although studies with super-resolution fluorescence imaging have been much more limited^6^.

Conventional quenched fluorescence microscopy has previously been demonstrated with the imaging of individual graphene oxide (GO) sheets, as a high throughput method to characterise their morphology^7^. Here we extend the technique to super-resolution imaging to characterize GO films with a much higher resolution (an order of magnitude improvement in resolution, from ~200 nm for diffraction limited techniques to 16 nm). Additionally, we demonstrate large advantages for STORM imaging of biological samples using GO coated substrates, in terms of improvements of the contrast and thus signal to noise on images due to the quenching of non-specifically bound fluorophores. The GO methods compare favourably with some other recent methods for background suppression in super-resolution fluorescence microscopy^6^. Our qSTORM technique is demonstrated with self-assembled peptides and pathogenic bacteria.

Resonance energy transfer (RET) is a mechanism that describes a non-radiative energy transfer process between a donor and an acceptor molecule. This process is strongly dependent on the donor-acceptor separation distance (*r*) and the efficiency decays with *r*^*−*6^ for two point fluorophores^8^. The effect has been found with a variety of acceptors and donors. A strong quenching (acceptor) effect of graphene was predicted by theory^9,10^ and observed experimentally^11^. The theory shows that the energy of a fluorophore can be transferred to the hexagonal lattice of graphene through a non-radiative mechanism which causes the quenching effect. The quenching effect is also possible with graphene oxide films, because they have the same hexagonal lattice as graphene and some similar electronic properties. According to calculations for graphene the quenching efficiency should have a *r*^*−4*^ dependence at long distances, where *r* is now the perpendicular distance between the graphene surface and a point fluorophore^12^. Comparison with the FRET (Förster RET) between two point fluorophores shows the graphene-point fluorophore system has a much larger energy transfer rate over the same separation distance and graphene is a much more efficient quencher than any currently known point fluorophore. Furthermore, compared with the traditional FRET process that tends to be wavelength specific, graphene shows a global response to fluorophores over a wide range of emission spectrum wavelengths^13–16^. Therefore the donors can be fluorescent dyes^16^, quantum dots^13^ or fluorescent polymers^17^ and they will all show a strong RET effect. Previous studies have thus shown that graphene and graphene oxide are excellent quenchers for a variety of fluorophores.

The combination of super-resolution fluorescence imaging with metal plasmonics is an active area of study^18^. Pure graphene substrates that demonstrate plasmonic behaviour have been used with STED^19^ super-resolution microscopies, but to our knowledge, have not been extended to graphene oxide (cheaper samples for scale up with a shorter, more convenient interaction length) nor has the contrast mechanism been rigorously quantified. Specifically, compared to pure graphene, graphene oxide is much cheaper, more transparent and easier to form films on glass surfaces using spin coating and could thus be conveniently scaled up in an industrial process.

In the current study the quenching effect of graphene oxide was studied using Stochastic Optical Reconstruction Microscopy (STORM)^1^. Specifically the dSTORM variant was used, since it conveniently only requires a single laser to excite the fluorophores to make them blink^20^. The method employs photoswitchable fluorophores to separate individual fluorescence signals by using the stochastic blinking of the fluorophores. The position of each localization was determined accurately from each frame by fitting a two-dimensional Gaussian to the point spread function (PSF) of each fluorophore’s signal. The final image was re-constructed by stacking all the localizations from each frame together with additional contrast provided by the fluorophores being extinguished by the strong RET effect when they are close to the GO. The resolution of the final re-constructed image can go down to 20 nm or even less^21^, and we demonstrated a resolution of 16 nm for the edges of the individual GO sheets. In addition we quantified the changes in the images’ resolution and contrast for both peptides and bacteria. The contrast of the images experienced large one or two orders of magnitude increases, whereas the resolution had a moderate improvement (a factor of 2.3 with the peptide fibres using the FOI metric).

## Experiment and Method

An aqueous solution of graphene oxide was made by a modified Humers’ method followed by a base wash process with 1 M of NaOH at 70 °C to further clean it^22,23^ (Supplementary Information S1). GO layers were spin-coated on a substrate from aqueous solutions. The speeds and durations of the spin-coating steps are listed in the Supplementary Information, S2. A densely packed graphene oxide layer of a thickness of up to 6 nm was created by using a GO aqueous solution with a concentration of 1.6 mg/ml and a randomly deposited sparse monolayer was made using a 0.03 mg/ml solution. The more dilute solution was used to observe the effect of single sheets of graphene oxide in a monolayer. The lateral size of individual graphene oxide sheets can be up to tens of microns and their thickness was around 1 nm, which made them well suited for observation with fluorescence microscopy. After coating, one hour of baking at 70 °C at standard pressure was applied to encourage the evaporation of any water molecules and improve the adhesion of the films. This improved the stability of the graphene oxide layer, especially in liquid environments (water or toluene). A round glass cover slip (diameter 15 mm and thickness No. 1.5) or a silicon wafer was used as a substrate. The substrate was sonicated in a 5% Decon solution for 30 min to clean the surface and ensure the surface was optimally hydrophilic to provide good adhesion of the graphene oxide sheets.

**Figure 1:**
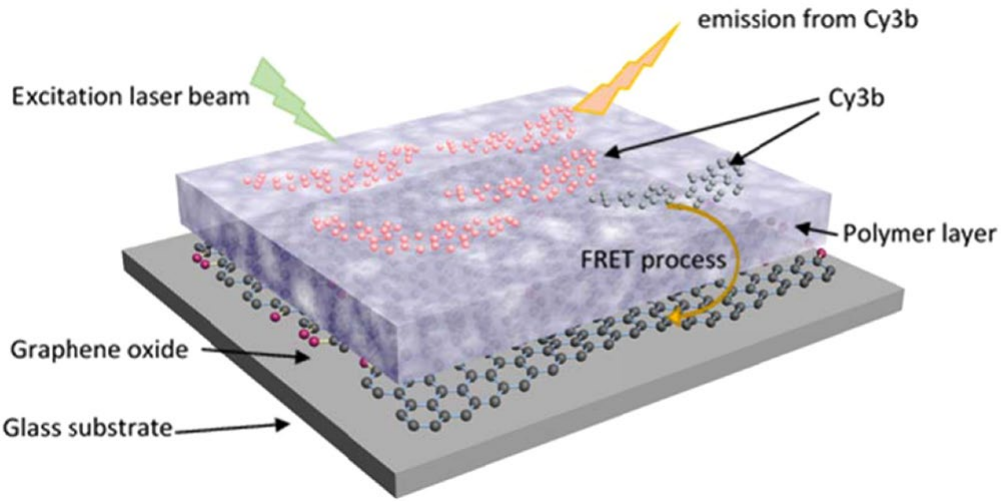
A schematic illustration of the stacked films (glass/GO/polymer/Cy3b) used to perform quenched Stochastic Optical Reconstruction Microscopy (qSTORM) on GO sheets. Cy3b fluorophores were coated on a polymer layer that had a well-defined thickness. RET quenching of Cy3b fluorophores provides a contrast mechanism that allows images of individual GO sheets to be reconstructed.

An additional layer was made of either polystyrene (for Cy3B) or poly(methyl methacrylate) (for Alexa Fluor 647) to separate the graphene oxide film and the fluorophores (Fig. 1). These hydrophobic polymers were chosen because they do not swell in the liquid environment and form a layer with a smooth surface. Both synthetic polymers are transparent and hydrophobic. Transparency ensures that the optical signal from the fluorophores can propagate through the polymer layer and be detected in the optical microscope. Hydrophobic surfaces help to prevent any liquid permeation, so that it minimizes any thickness changes in aqueous environments. The polymer spacer layers were spin-coated on the top of the graphene oxide layer from the corresponding toluene solution at a speed of 3000 rpm for 50 seconds. The gap distances were controlled in the range 2–15 nm by varying the polymer concentration. Thickness measurements were performed with a spectroscopic ellipsometer (*J.A. Woollam Co., Inc*. E-2000) on silicon wafers (the parameters of the optical model used for ellipsometry fits are listed in the Supplementary Information, S3). When preparing the sample on transparent cover slips, exactly the same process was used as on the silicon wafer to obtain a layer with the same thickness.

The two fluorophores used were either Alexa Fluor 647 (AF 647) (mean excitation/emission wavelength 650 nm/665 nm respectively from Thermo Fisher Scientific) attached to bovine serum albumin (BSA) or unconjugated Cy3B NHS ester (mean excitation/emission wavelength 559 nm/570 nm respectively from GE Healthcare Life Sciences). The Cy3B was used unconjugated because the π-stacking interactions^24^ between the benzene rings in the dye molecules and the benzene rings in polystyrene created an attractive interaction and thus encouraged strong adsorption.

Stacked systems of films of graphene oxide, synthetic polymer and fluorophore were studied via quenched Stochastic Optical Reconstruction Microscopy (qSTORM). The stacked structure is illustrated in Fig. 1. To optimize the performance of the fluorophores, different image buffers were used for AF 647 and Cy3B to reduce oxidation and thus photobleaching. A Gloxy buffer was used for AF 647^25^ and a OxEA buffer was used for Cy3B^26^. The recipes of the two imaging buffers are listed in the Supplementary Information, S4.

A sample of self-assembled I_3_K peptide fibrils was used to demonstrate the quenching effect of sparse single layer graphene oxide sheets. The fibrils were aged for a week to achieve steady state structures and then labelled with Cy3B-NHS Ester fluorophores. STORM can thus be used to study the self-assembly of aggregates of the surfactant-like peptide I_3_K^27^. The quenching effect of dense packed graphene oxide layers was also studied using *E. coli* samples. The GO sheets we used had been carefully washed to remove sulphated impurities. Previous careful experiments indicate there is a negligible bactericidal effect for GO on *E. coli* and *Staphylococcus aureus* bacteria^28^. The GO coated coverslips we created present negatively charged hydrophilic surfaces fairly similar in character to the underlying SiO_2_ cover slips traditionally used for imaging biological cells and they thus have a good biocompatibility. An additional polylysine layer was used to immobilise *E. coli*, but it was a matter of convenience to immobilize the bacteria and was not an issue of bactericidal effects of the underlying GO layer. Specifically, 1 mL of 0.01% w/w poly-L-lysine solution (M_w_ 150K-300K from Sigma-Aldrich) was put on the surface for 10 minutes and then the surface was rinsed with UHQ water to remove unattached polymer. The bacterial capsules thickness was in the range 200–400 nm due to large membrane-bound lipopolysaccharide brushes on the bacteria’s surfaces and it was labelled with a primary antibody (mouse IgG anti-K1^29^ antibody) at 1:400 concentration in 1% BSA and a secondary antibody (anti-mouse F(ab′) Alexa Fluor 647) at 1:1000 concentration in 1% BSA. The length of each bacterium was around 2 µm and their width was 1 µm.

In our experiments a Digital sCMOS (scientific CMOS ORCA Flash 4.0 v2) camera (Hamamatsu C11440-22CU) was used to capture diffraction limited images at 100 frames per second and the rest of the STORM apparatus was as described previously^27,30^. A 100x objective lens (UAPON 100XOTIRF, Olympus) with a Numerical Aperture (NA) of 1.49 was used. The TIRF objective lens was used with standard fluorescence microscopy illumination i.e. TIRF images were not formed. To re-construct one super-resolution image, 10000 diffraction limited images were collected. The laser power was set at 70 mW and the laser power density measured from the objective lens was 100 mW/cm^2^. The lasers were used to both activate and excite the fluorophores (the dSTORM technique). Sample drift was eliminated using a MadCity Labs C-focus system that adjusted the *z* focus of the objective lens using feedback from a magnetic transducer. Coherent artefacts from the laser were suppressed using a speckle scrambler (the light was launched into the microscope using an optical fibre that was oscillated with a mobile phone motor). An ImageJ plugin, ThunderSTORM^31^, was used to analyse the image series to create super-resolution images. Details of the parameters used in the image analysis are available in the Supplementary Information Section (S5).

## Results and Discussion

**Figure 2:**
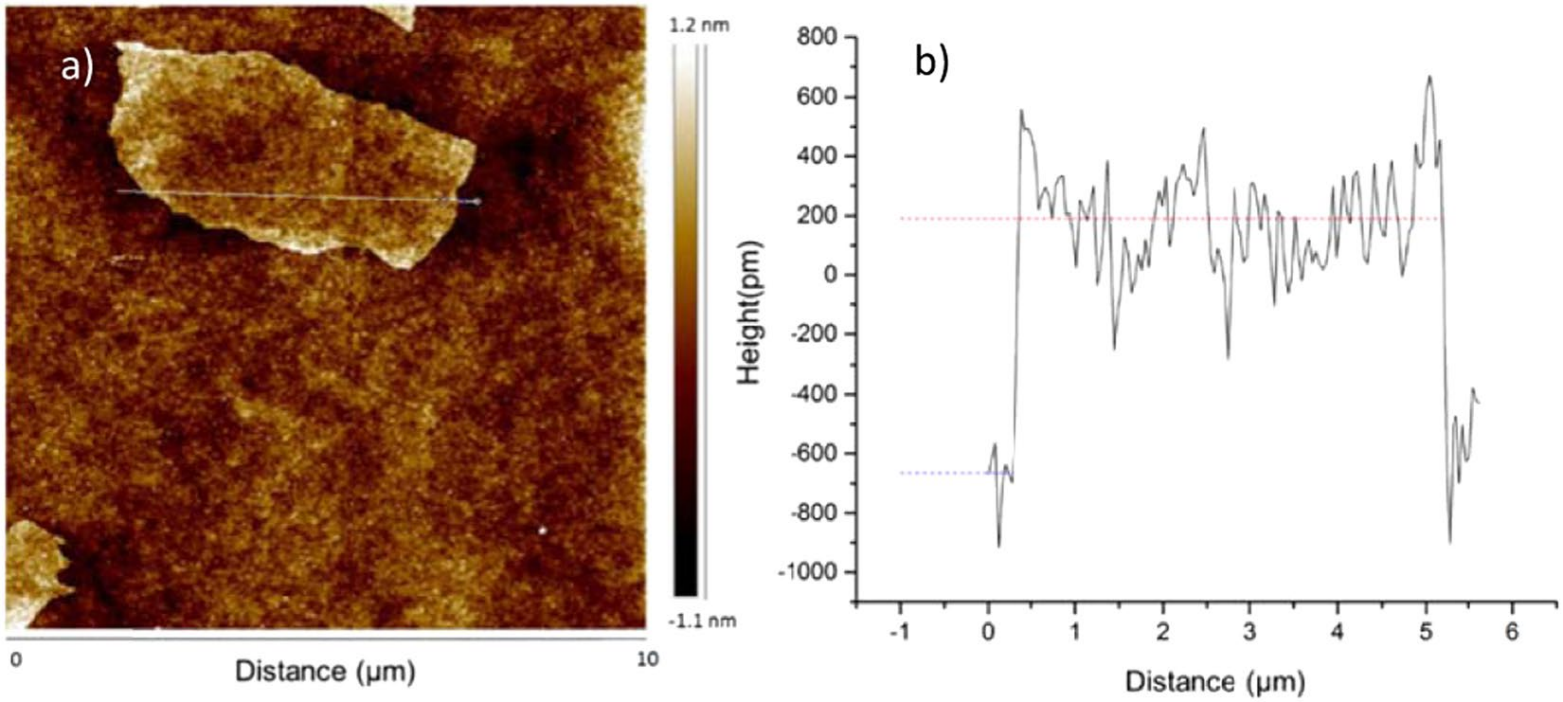
(**a**) An atomic force microscopy (AFM) image of a GO monolayer deposited on a glass surface. (**b**) The corresponding height scan as a function of lateral distance along the line indicated in a).

Initially single sheets of graphene oxide were studied. Figure 2 is an atomic force microscopy image of a sparse monolayer of single graphene oxide sheets deposited on a glass slip. The height plots across the graphene oxide sheets indicated that the average height of the sheet was about 0.8 nm which means it consists of a monolayer of graphene oxide^32,33^. The lateral sizes of single layers of graphene oxide were in the range of a few microns to tens of microns.

A polystyrene (PS) layer was spin-coated onto the graphene oxide coated surfaces from a toluene solution. The composite layers were then baked for 20 hours to anneal the PS layer. Finally the polymer surface was coated with an aqueous solution of Cy3B dye NHS ester (100 ng/ml) and then rinsed with deionised water to remove free dye molecules.

**Figure 3:**
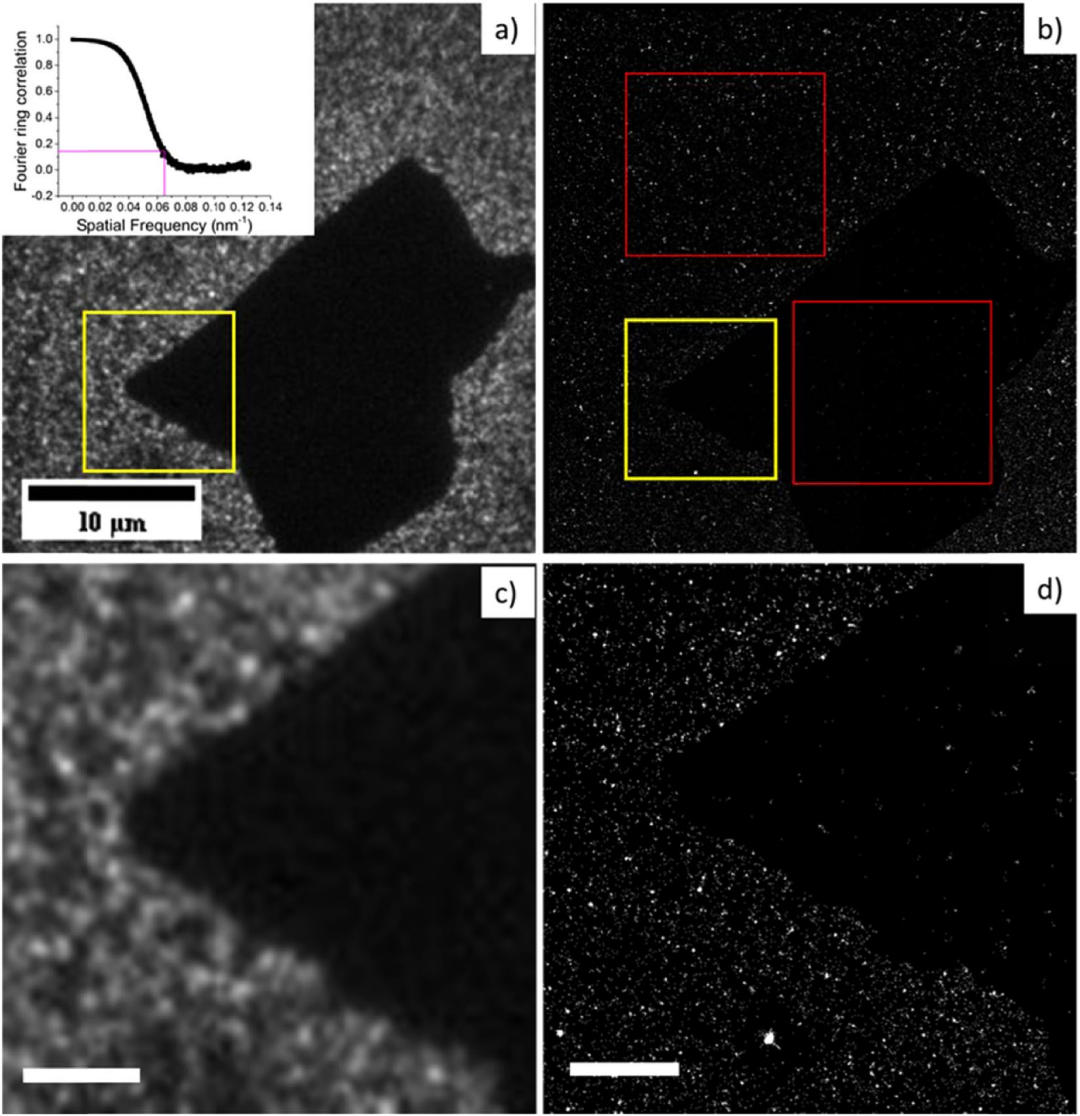
(**a**) A diffraction limited fluorescence image of a single sheet of graphene oxide deposited on a glass substrate with Cy3B fluorophores. A polystyrene layer with a thickness of 2 nm was used as a spacer layer. (**b**) A super-resolved fluorescence (qSTORM) image of the same graphene oxide sheet. The red rectangles label the area used to calculate the average photon density. (**c**) A zoomed in region (yellow rectangle from the diffraction limited image shown in (**a**). (**d**) A zoomed in region (yellow rectangle) of the super-resolution graphene oxide image. The inset in (**a**) is a Fourier ring correlation of the super-resolution image as a function of spatial frequency calculated from image (**b**)^49^. The standard threshold for the correlation to calculate the resolution is defined as 1/7 and this gave a resolution of 16 nm for the image shown in both (**b**,**c**).

**Figure 4:**
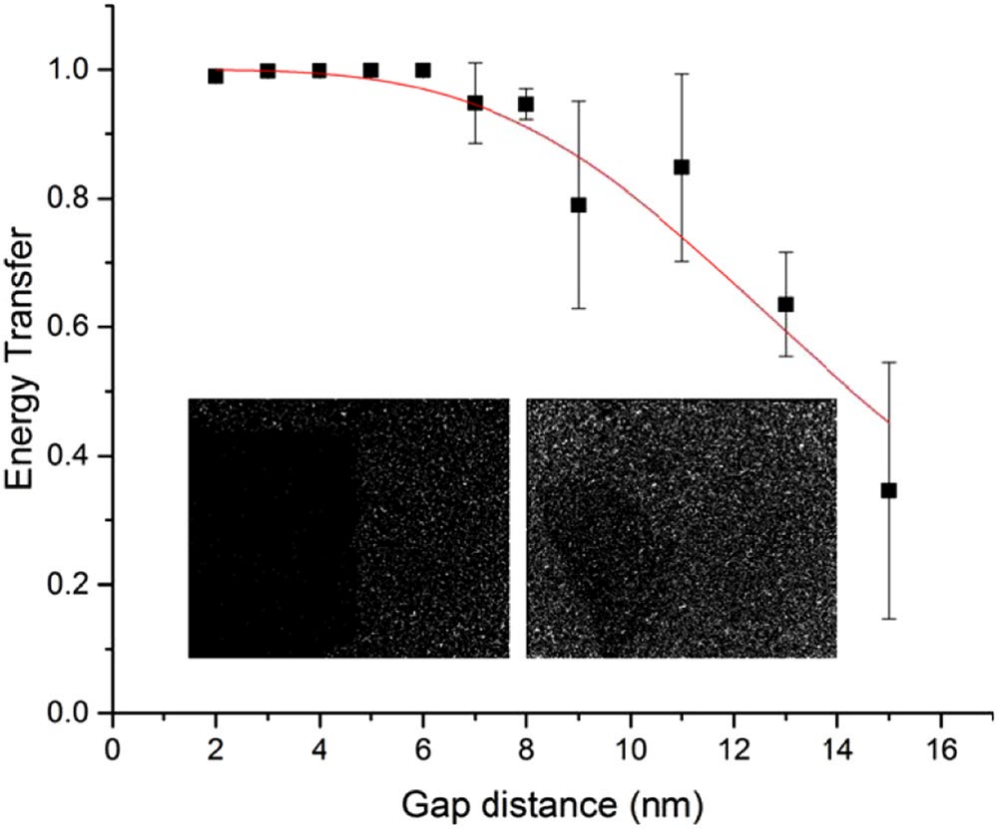
The energy transfer efficiency calculated from qSTORM images of graphene oxide as a function of different polystyrene substrate layer thicknesses (2–15 nm) with Cy3B (the gap distance). The Red curve was the power law fit of equation (4). Inset images were re-constructed using qSTORM with graphene oxide monolayers. The polystyrene spacer thicknesses for the qSTORM images were 2 nm (left) and 13 nm (right).

Figure 3(a) shows a diffraction limited image of the quenched fluorophores above the graphene oxide sheet. Figure 3b shows a super-resolved image of a GO sheet created using dSTORM and Fig. 3c,d show zoomed regions (enclosed with yellow rectangle) within Fig. 3a,b to demonstrate the improved resolution. A sharp edge with well resolved rough structures in Fig. 3d is obtained compared to the blurred boundary in Fig. 3c. The image resolutions can be calculated using the Fourier Ring Correlation method^34^ (inset Fig. 3a i.e. when it falls to a value of 1/7) and it is found to be 16 nm for the image shown in Fig. 3b,d. This is an order of magnitude better than possible with diffraction limited fluorescence microscopy i.e. ~200 nm^7^. There is no standard literature value for the light absorption of a single layer of graphene oxide. The visible light absorption of monolayer graphene is well studied and it absorbs 2.3% of the total light that passes through a sheet^35^. An aqueous solution of reduced graphene oxide/graphene shows a much higher absorption than graphene oxide at the same concentration^7,36^. The transmittance of a 20 nm GO film has been previously measured to be ~20%^37,38^. This implies that the graphene oxide monolayer (thickness ~1 nm) will absorb less light than 0.45% of an incident visible light source. The absorption spectroscopy peaks of both graphene and graphene oxide solutions occur at 200–300 nm, so the absorption at larger wavelengths (>400 nm) should be less than 0.45% for GO^39^. Therefore, the super-resolved shadow in Fig. 3b is not due to the absorption of light by the graphene oxide. The main reason for the high contrast is the quenching of fluorophores above the graphene oxide covered region. According to theoretical predications, an efficient quenching effect is expected when a graphene oxide sheet is close to the fluorophores^9,10^. This means that the fluorophores tend to transfer energy to the graphene oxide sheets via RET instead of emitting a photon. The quenching effect can be calculated experimentally using re-constructed super-resolution images. For each fluorophore localization, the integral of a fit of a Gaussian point spread function gives the number of photons emitted. The ratio of photon densities can be used to evaluate the contrast from the graphene oxide area compared to the non-graphene oxide area. In Fig. 3b), the red rectangles enclose two areas. The total photon number in the graphene oxide covered area was 1667 with an average intensity of 1817. The emitted photon density was calculated to be 2.3 × 10^4^ *photons*/*μm*^2^. Then the photon density of the non-graphene oxide covered area was found to be $107\times {10}^{4}\,photons/\mu {m}^{2}$. The photon density of the graphene oxide covered area and the non-graphene covered area are labelled *I*_*GO*_ and *I*_*NGO*_ respectively. The energy transfer efficiency (*ε*) between graphene oxide and dye molecule was evaluated using $\varepsilon =1-\frac{{I}_{GO}}{{I}_{NGO}}$ ^40^. The performance of fluorophores also depends on a variety of other parameters, such as the imaging buffer, the laser intensity, or the time the sample had been exposed to light, so the energy transfer was always calculated from the same image, since *I*_*GO*_ and *I*_*NGO*_ are self-referenced and do not need to be corrected. For each gap distance, 5 images were taken from different places on the same sample. The energy transfer was extracted individually from each image and the mean value was calculated. The energy transfer is plotted against the polymer spacer thickness in Fig. 4. The number of localizations within GO is much less than the number of localizations outside GO due to the quenching effect. The uncertainty, sigma and photon number per localizations plot within/outside GO area are provided in the Supplementary Information, S6. We believe this self-referenced method compares favourably with more complicated pulsed laser techniques based on the lifetime of fluorophores^12^.

Figure 4 shows that the energy transfer from single GO sheets decays as the thickness of the polystyrene layer increases. The plot is the mean value of 5 measurements at each gap distance and the error bars were calculated from the corresponding standard deviations. The two insets show re-constructed qSTORM images at polystyrene spacer thicknesses of 2 nm and 13 nm respectively. When the polymer spacer thickness is small, the graphene oxide sheets are obvious in the images and they have a high contrast. The graphene oxide quenches not only the fluorescent dye molecules, but also any other auto-fluorescent molecules in the environment that are surface adsorbed or very near to the surface. The quenching efficiency decreases with the increase of the polymer spacer thickness. The error bars are smaller than the data points in Fig. 4 when the spacer thickness is less than 7 nm. The quenching efficiency is very close to 1 in this range so the variation is not significant. The error bars become more obvious as the thickness increases and the non-radiative decay rate becomes comparable with the radiative decay rate. The reason for this variation is that the graphene oxide sheets are not perfectly flat and there are thickness variations of the spin coated polystyrene layer. Since the decay rate changes rapidly with the gap distance, a small variation in gap distance will cause a huge change in the decay rate. The AFM image (Fig. 2b) shows that the height scan of a region of GO has a variation of 0.8 nm and previously reported SEM^41^ images also show wrinkled structures for graphene oxide sheets. This uneven surface implies heterogeneous thickness changes across the GO surface. The wrinkled areas of graphene oxide will quench the fluorophores more effectively than an idealized monolayer. Therefore these areas will quench the fluorophores more efficiently than is expected. Wrinkled morphologies are more important when the rate of non-radiative decay is comparable to the radiative decay of the dye molecules. When the spacer thickness reached 13 nm, the quenching effect could only be barely seen with the naked eye on re-constructed images. When the spacer thickness was increased further, the quenching effect further decreased until it was eventually undetectable (>15 nm). Theoretical predictions for the rate of energy transfer by RET with a point-like fluorophore and a pure graphene sheet were first given by Swathi and Sebastian^9,10^. They found that the quenching efficiency (*ε*) obeys a *r*^*−4*^ dependence (*r* is the distance between the fluorophore and the graphene sheet). Later Gomez-Santos and Stauber extended the calculation^42^ and gave a relation between the energy transfer efficiency (*ε*) and the gap distance (*z*) (more details of the derivation are in the Supplementary Information, S7):

1 $$\varepsilon =1-\frac{{I}_{GO}}{{I}_{NGO}}=1-{[1+A{(\frac{\lambda }{z})}^{4}]}^{-1}$$

where *I*_*GO*_ and *I*_*NGO*_ are the photon densities of the graphene oxide covered area and the non-graphene oxide covered area respectively^43^. The variable *A* was the single fit parameter for the model and was found to be 2.6 ± 0.4 × 10^−8^ (no units). It contains an orientation factor and a coupling coefficient. The structural differences between graphene and graphene oxide are also incorporated in *A*.

At very small gap distances the energy transfer efficiency is very slightly less than 1 (Fig. 4). The discrepancy with respect to the prediction of equation (1) is negligible with our current data set, but it may be due to inter-fluorophore FRET between fluorophores in surface adsorbed aggregates which is not accounted for in equation (1).

The form of equation 1 implies the quenching effect linearly depends on the emission wavelength of a fluorophore, but has a *r*^*−4*^ spatial dependence, where *r* is the distance between a fluorophore and the graphene surface. Considering most commonly used fluorophores have an emission wavelength of a few hundred nanometres, the quenching effect is generic and will occur with most fluorescent dyes. A second fluorophore, Alexa Fluor 647 (Thermo Fisher Cat A34785), was also used with the same experimental setup to image single GO sheets. The fluorophore was conjugated to Bovine Serum Albumin (BSA), which can adsorb to Poly(methyl methacrylate) (PMMA) via a hydrophobic interaction. BSA globules adsorb strongly in aqueous solutions and they are used as a standard blocking agent in molecular biology experiments e.g. they are used in commercial pregnancy kits^44^. An example of a re-constructed image of a single graphene oxide sheet using fluorescently conjugated BSA is available in the Supplementary Information S8 and it is similar to those measured with Cy3B (Fig. 3).

The global quenching ability of graphene oxide layers can therefore be used to remove background noise in fluorescence experiments with a range of potentially useful substrate layers, such as polystyrene, PMMA, and polylysine (to be shown later).

The non-specific binding of fluorophores often causes high backgrounds during fluorescence imaging of biological materials and soft condensed matter. With more fluorophores to label the sample there will be a stronger image signal, but this comes hand in hand with increased background noise and reduced contrast, so it is difficult to balance the fluorophore concentration used. Excess dye molecules tend to diffuse and then bind to the substrate and the typical size of a fluorescent dye molecule is 1–2 nm. The total size of a dye molecule conjugated to secondary antibodies, which are often used to label specific biological structures, is on the order of 10 nm. Thus the background fluorescence noise can be quenched efficiently by an additional graphene oxide layer underneath a sample (Fig. 4), since it provides strong quenching at thicknesses ≤15 nm. Historically biologists have predominantly used blocking agents (e.g. BSA) or counter stains (e.g. Evans Blue) to counteract problems with non-specific binding (it is also a big issue in antibody based diagnostic tests e.g. pregnancy kits)^44^, but these methods are time consuming and do not provide optimal contrast. GO coated cover slips are a more effective solution to this problem.

**Figure 5:**
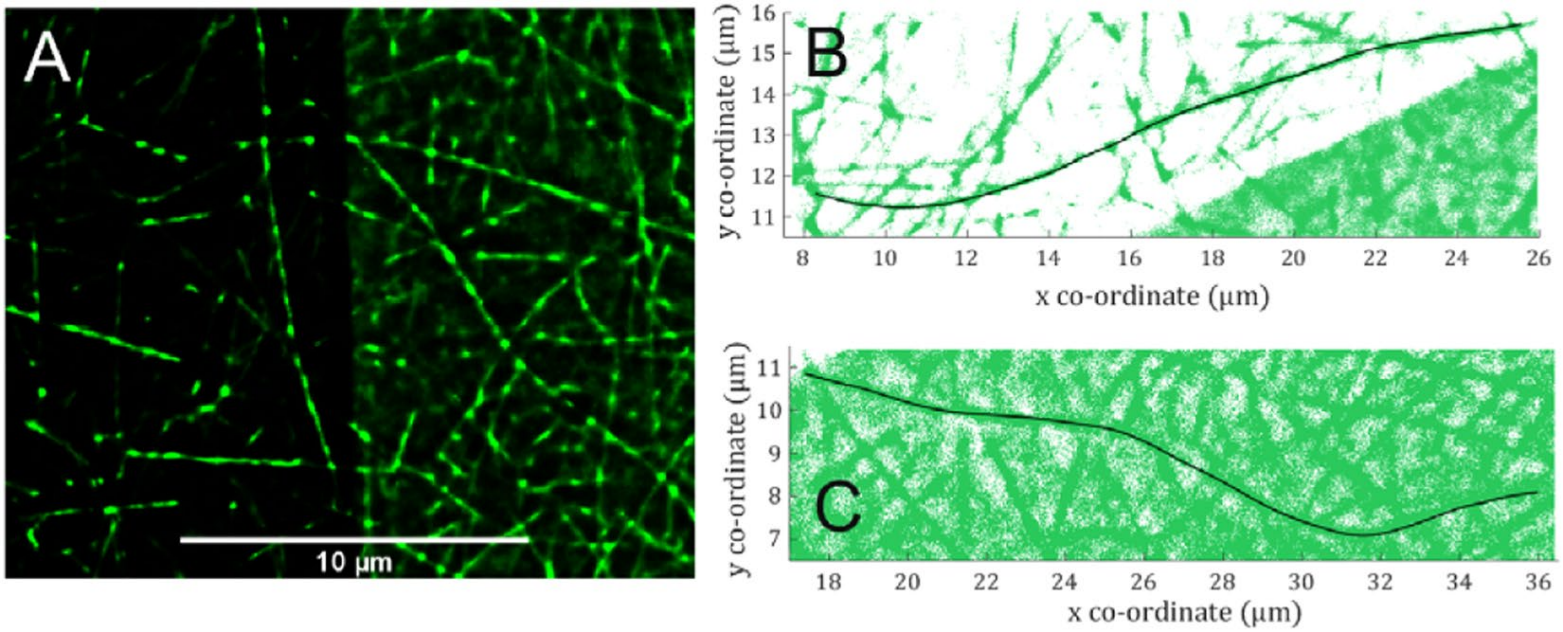
(**a**) STORM reconstruction of I_3_K peptide fibrils at the solid/liquid surface. On the left of the image a graphene oxide flake is present. The graphene oxide effectively reduces the background signal due to surface adsorbed free fluorophores and fluorescently labelled peptide monomers. (**b**,**c**) Show the contours of peptide fibres located using image analysis software (black lines, FiberApp)^47^. The surrounding localisations of two example fibrils can be compared where graphene oxide is present (**b**) and where it is not (**c**). The presence of graphene oxide allows for potentially more accurate analysis using snake based algorithms and reduces ambiguity when determining localisation distributions along a fibril. The sample preparation and image analysis was identical to that presented in a previous work with the addition of a GO coated substrate^28^.

Figure 5 shows STORM images of self-assembled I_3_K peptide fibrils prepared in pure water at 10 mM peptide concentration. Surface imaging of self-assembled fibrils is an active area of research for amyloid diseases, *de novo* materials and as anti-microbials. Previous investigations have studied the exchange of peptides between fibrils and their self-assembly mechanisms^45,46^. Due to the dynamic nature of self-assembled peptide structures all unnecessary sample disturbance needs to be minimised. Therefore, removal of unconjugated fluorophores was not performed when imaging I_3_K fibrils, since washing could damage the delicate structures formed. The large concentration of the unconjugated fluorophores and the fluorescently labelled peptide monomers could readily adsorb to all the exposed surfaces, and generated a significant noise in the STORM images. In the image shown, the left side contains a graphene oxide flake between the fibrils and the cover slip, which effectively quenches noise due to fluorophores adsorbed non-specifically to the surface. Fibril identification in STORM images using image analysis algorithms can be challenging and by reducing the background noise to a very low level the graphene oxide layer offers the prospect of more accurate analysis. Furthermore, the analysis of localisation distributions in STORM images is becoming a popular method for investigating mixtures of monomers within self-assembled structures^34,46^. These methods rely upon the accurate counting of the number of monomers within a structure, which is based upon the counting of localisations. If there are a significant number of localisations present in the background around the fibres that provide a large background noise, this will introduce an ambiguity into the measurement which is hard to quantify. Figure 5 shows two examples of segmented fibrils (dark continuous lines on B and C created using FiberApp)^47^; one in the region containing graphene oxide (B) and the other in the region without graphene oxide (C). The graphene oxide has removed almost all the background localisations due to a small amount of surface adsorbed fluorescent dye or labelled peptide monomer; this enhancement in raw data quality has the potential to greatly improve results from these kinds of samples. Specifically, the image contrast was calculated ($C=\frac{I-{I}_{b}}{{I}_{b}}$, where *I* is the average intensity along a peptide and *I*_*b*_ is the background intensity) and a 10 ± 6 times improvement in *C* was observed with/without GO for the images shown in Fig. 5. The contrast changes from 13 (±47%) to 133 (±40%) with graphene oxide. Furthermore, the resolutions of re-constructed images in both cases (with and without graphene oxide) were determined using the Fourier Ring Correlation method^48^, SI S9, Fig. S2. The FRC resolution of the image with graphene oxide is 19 nm, while the resolution without graphene oxide is 23 nm. There is thus a slight improvement in the resolution with the graphene oxide layer. The average uncertainty of localizations is about 18 nm in both cases, i.e. with/without GO (the uncertainty plot is shown in the Supplementary Information, S8), which contributes to the observed resolution.

**Figure 6:**
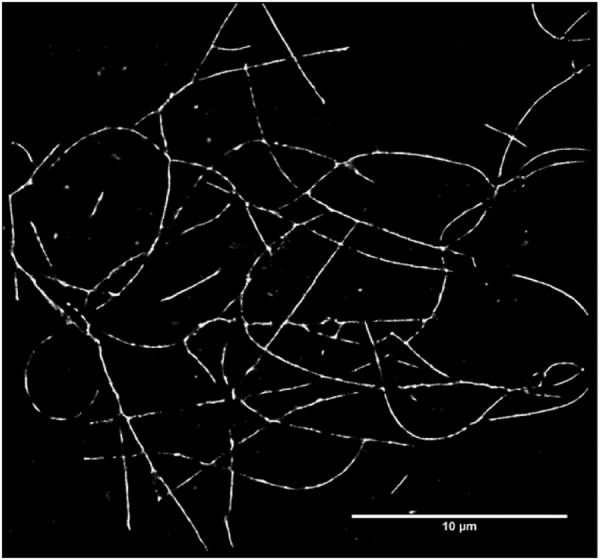
A reconstructed STORM image of I_3_K peptide fibres is shown. A graphene oxide film of 5 nm thickness was used (compared with the single GO flake shown in Fig. 5) and a significant improvement in resolution was observed according to the FOI metric.

Much of the background noise is quenched by a monolayer GO sheet, but an even more efficient reduction in noise can be achieved using a thicker GO film with multiple layers. Figure 6 is an *on-surface* super-resolution image of the self-assembled I_3_K peptide fibre on a 5 nm GO film. The *on-surface* images of self-assembled peptide contain large levels of noise due to the non-specifically bound fluorophores e.g. in Fig. 5. With the help of graphene oxide, we are able to produce *on-surface* images with comparable contrast to the *off-surface* (bulk) images of I_3_K peptide fiber to those we previously demonstrated^28^. The background noise is not due to a transmittance problem since the transmission of a 5 nm GO layer is calculated to be 2%^37^ (which compares favourably to a similar thickness of graphene which would have 11% absorption)^49^.

**Figure 7:**
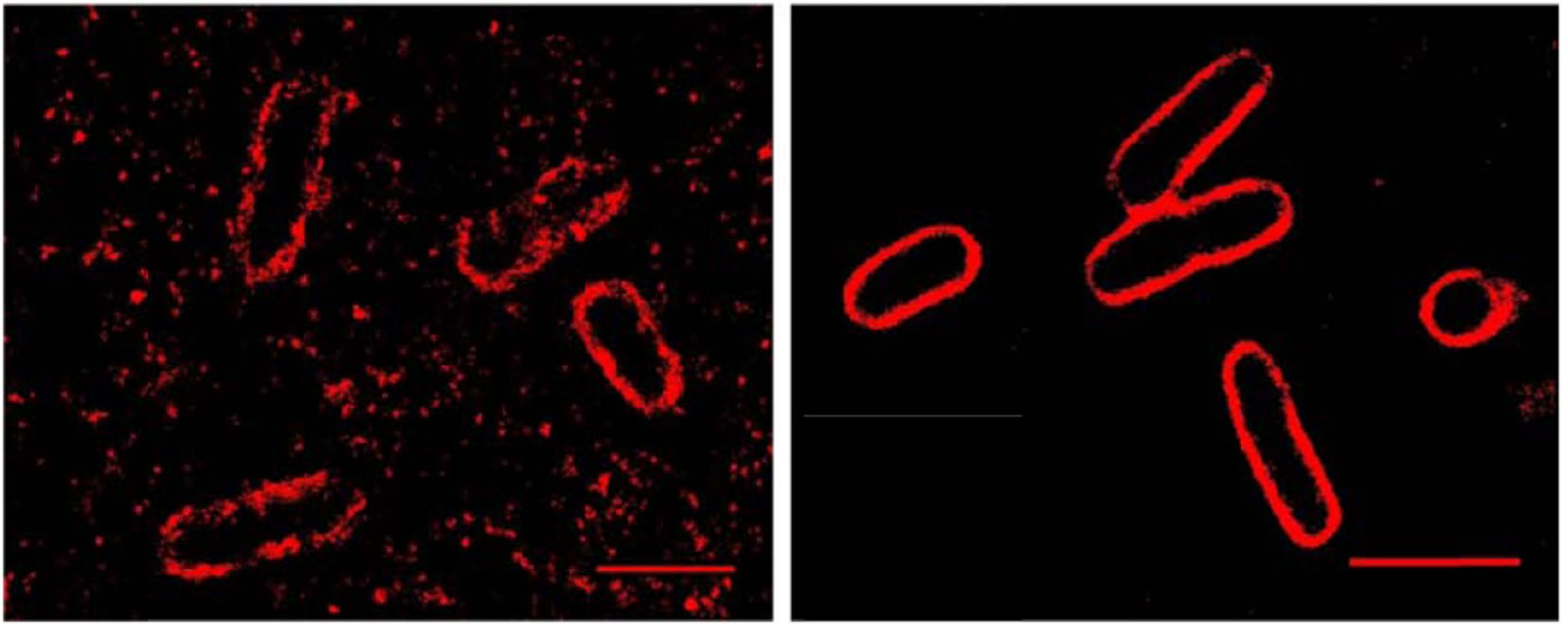
A reconstructed STORM image of *Escherichia coli* bacteria (strain EV36) on a standard glass substrate (left) and on a 5 nm film of graphene oxide coated on a standard glass substrate (right). The capsular structures (200–400 nm) on the outside of the pathogenic bacteria have been labelled with antibodies connected to Alexa Fluor 647 fluorophores. Capsules are a key mechanism of antibody resistance and increase the virulence of bacterial pathogens^50^.

Figure 7 shows encapsulated *Escherichia coli* bacteria, strain EV36, grown at 37 °C in Luria-Bertani (LB) broth up to the mid-log growth phase, with an optical density at 600 nm (OD_600_) of 0.5. With a 5 nm layer of graphene oxide the non-specifically bound fluorophores were significantly quenched. An image with a much lower background was achieved, because the background due to non-specifically bound antibodies and any other surface adsorbed autofluorescent molecules (e.g. proteins from the bacteria) has been eliminated. Since the efficient quenching range (<15 nm from Fig. 4) was much less than the diameter of the *E. coli*, the quenching effect on signals from the actual sample was negligible. The images in Fig. 7 are from slices through the bacteria, approximately 500 nm from the GO coated surface. The image contrast (*C*, calculated for the capsule images compared with the background intensity) experienced a 138 ± 63 times improvement with/without GO. The contrast changes from 24 (±27%) to 3317 (±37%) (arbitrary units) with graphene oxide. The FRC resolutions with and without graphene oxide were 55 nm and 58 nm respectively (the Fourier Ring Correlation plot is shown in the Supplementary Information, S10, Fig. S5) i.e. again there is a slight improvement in image resolution with the GO coating. The mean uncertainty of each localization is 18 nm without the GO layer and it increases to 24 nm when the GO layer is present (the uncertainty plot is shown in the Supplementary Information S10, Fig. S5). This change in localization uncertainty is due to the slightly reduced number of photons from the sample with the GO layer, but it is still within an acceptable range and does not adversely affect the image resolution (which is still improved due to improved contrast). Since a thicker layer of graphene oxide was used, the changes in localization uncertainty are more obvious than with the case with a single layer graphene oxide sheet. The effective resolution in STORM images^50^ is a complex mixture of fluorophore localization uncertainty ($\sim \sqrt{N}$, where *N* is the number of photons), the labelling density and the background signal to noise (related to the contrast). All three parameters need to be optimised to provide optimal image resolution. With the peptide and bacteria samples that we imaged with qSTORM the resultant image resolution was slightly improved with GO according to the FRC metric, whereas there was a substantial improvement in contrast by two orders of magnitude.

Theoretically it is clear that contrast and resolution are related according to the Rayleigh definition of resolution for an optical instrument i.e. the resolution is when the contrast between two point spread functions reaches 26.3%^51^ (and a similar relationship is expected with stochastic extensions of this definition)^52^. Therefore large improvements in resolution might be expected if there are large improvements with the contrast. However, with the FRC resolution metric only modest improvements in resolution are observed. We believe this apparent contradiction in our results is due to the inability of the Fourier Ring Correlation method to separate the effects of aggregated fluorophores (an artefact removed by the GO coatings) from the actual signal (simulations are included in SI 11). We thus believe that GO coatings improve the image resolution, but the effect is not well captured by the FRC technique. Based on the FRC methods the improvements in the resolution are 21% and 5% for the peptides and bacteria respectively. These improvements are relatively modest (on the border of statistical significance), but could be improved upon in future studies, since they are sample and metric dependent. However, particularly useful in the current study is the demonstration of the removal of aggregated fluorophore artefacts by the GO coatings, which may be better characterised by a χ^2^ error analysis if the ground truth images are known.

**Figure 8:**
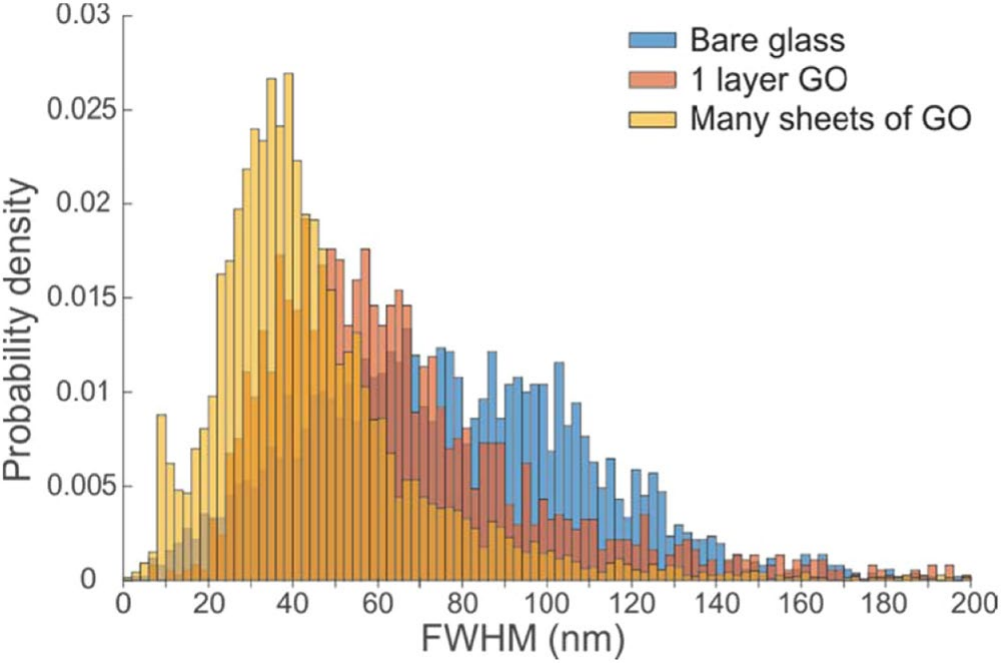
Histograms of the FWHM of the widths of peptide fibres at regularly placed positions along their backbones (determined using FiberApp)^47^. The width distribution shifts to smaller values in the order 5 nm graphene oxide (many sheets) <1 layer of graphene oxide < no layers of graphene oxide. The width of the effective point spread function is equal to the FWHM minus 11 nm (the fibre width from AFM)^27^ minus 1 nm (the size of the fluorophore).

An alternative method to quantify the resolution follows what we call the *feature of interest* (FOI) technique. Here the effective point spread function (PSF) of the images is calculated based on the segmentation of the images into regions that are classified as either feature or background. Thus the peptide images were segmented into either peptide or background using Fiberapp^47^ and statistical analysis of cross-sections through the diffraction limited fibres were fitted with Gaussians (the fibres are known to be 11 nm in width)^27^ to provide the width of the effective point spread function i.e. the full width at half maximum minus the fibre width minus the fluorophore size, FWHM −11 nm – 1 nm. Results of this Gaussian width analysis are shown in a histogram of fibre thicknesses (Fig. 8). A dramatic improvement in resolution using the graphene coatings is now observed using the FOI metric. The width of the effective PSF (modal value) is 56 nm for no coating, 38 nm for single graphene oxide flakes and 24 nm for a 5 nm GO coating. The FRCs are seen to underestimate the resolution compared with the FOI method when there are large aggregates of non-specifically adsorbed fluorophore. The FOI methodology lends itself to automation within a machine learning framework (containing the strong Bayesian assumption of the segmentation prior). To apply the FOI method to the capsule images would require more extensive calculation, since a challenge is to separate the effect of the capsule thickness from the effects of both the PSF and the capsule roughness (all three effects are convolved together). The FOI metric does provide additional evidence that there is an improvement in resolution with GO coatings and in the case of peptide fibre diameters it is fairly significant (a factor of 2.3 improvement).

We expect the improvement in contrast due to the GO films can be extended to a wide range of fluorophores, fluorescently labelled samples (e.g. it would be good to test the method with eukaryotic cells, such as human cancer cells) and fluorescent microscopy methods. The only study of STORM with graphene in the literature^53^ was a correlative study with SEM and did not discuss the favourable properties of graphene for contrast enhancement or resolution enhancement i.e. graphene was only used as a thin transparent substrate. We have demonstrated that RET contrast enhancement is a key property of graphene oxide (and it is also expected for graphene) coated substrates in optimizing images and GO is more convenient than graphene in terms of cost and ease of preparation.

## Conclusion

Super-resolution images of single sheets of graphene oxide were obtained via qSTORM. The rough edges of graphene oxide sheets were imaged with a resolution of 16 nm; a two orders of magnitude improvement on previous diffraction limited fluorescence microscopy methods^7^. The distance dependence of graphene oxide quenching of fluorophores was studied in the range 2–15 nm and it is believed to be due to the non-radiative resonance energy transfer between graphene oxide sheets and the fluorescent dyes i.e. RET. Sophisticated optical techniques have been previously used to eliminate backgrounds with other super-resolution fluorescence microscopy methods e.g. STED^6^. Our methodology compares favourably with previous methods, since it can be easily adapted to a variety of fluorescence microscopy methods (we have demonstrated it with STORM and diffraction limited fluorescence microscopy, but it could be extended to STED, PAINT^54^, selective plane illumination microscopy, structured illumination microscopy and confocal microscopy, since for surface bound specimens it will extinguish some background fluorescence artefacts, improve the contrast and may lead to a modest improvement in resolution e.g. a factor of 2.3 was demonstrated with the peptides. Compared with using graphene, GO is cheaper, absorbs less light and is easier to coat on a substrate surface. An additional advantage of qSTORM is the ability to study submerged GO films, which is not possible with scanning electron microscopy and would be useful to study surface enrichment in GO/polymer composite materials that have high performance mechanical properties^55^. A big improvement of signal to noise ratio was also achieved in qSTORM experiments by an additional graphene oxide layer placed underneath both live bacterial cells and peptide samples to quench background noise (two orders and one order contrast improvements respectively). According to the FRC metric the image resolution experienced a slight improvement at 19 nm and 55 nm respectively i.e. 5% and 21% relative improvements with/without GO. However, using the feature of interest metric a much more substantial improvement was measured at 230% with the peptides. The disagreement is thought to be due to how the metrics handle large fluorophore aggregate artefacts and the FOI method is superior in this respect. A variety of polymer layers were demonstrated to separate graphene oxide layers from the sample to provide optimal surface chemistry for the support of the sample, while quenching the fluorescent background. Future work could improve the roughness of GO layers and thus allow more accurate height measurements based on RET in the 1–20 nm range. Other fluorescence based techniques could also benefit from background suppression using GO e.g. flow cytometers or high throughput microfluidics measurements with GO coated channels^56^.

## Electronic supplementary material

Supplementary Information
