# Supplementary material for: Quenched Stochastic Optical Reconstruction Microscopy (qSTORM) with Graphene Oxide
Source: Sci Rep. 2018 Nov 16;8:16928. doi: 10.1038/s41598-018-35297-4 (PMC6240082; doi:10.1038/s41598-018-35297-4)
Supplement: Supplementary file 1 — Supplementary Information [file 41598_2018_35297_MOESM1_ESM.pdf]

SUPPLEMENTARY INFORMATION SECTION

Quenched Stochastic Optical Reconstruction Microscopy (qSTORM) with Graphene Oxide

Ruiheng Li[1], *Pantelis Georgiadis*[1,2], *Henry Cox*[1], *Sorasak Phanphak*[1,3], *Ian Roberts*[3], *Thomas A. Waigh*[1,2*], *Jian R. Lu*[1⊥]

[1]Biological Physics, School of Physics and Astronomy, University of Manchester, Oxford Rd., Manchester, M13 9PL, UK.

[2]Photon Science Institute, University of Manchester, Oxford Rd., Manchester, M13 9PL, UK.

[3]Division of Infection, Immunity and Respiratory Medicine, Michael Smith Building, Oxford Rd., M13 9PT, UK.

[*]t.a.waigh@manchester.ac.uk

[⊥]j.lu@manchester.ac.uk

S1. Graphene oxide aqueous solution preparation

The Hummers' method provides a simple straightforward route to prepare GO suspensions[1]. Some modifications were made to the original method to make the process safer and quicker. Firstly, potassium permanganate ($KMnO_4$ 6 g) was slowly added into a mixture of graphite flakes (2 g), sodium nitrate ($NaNO_3$ 1 g) and sulphuric acid (H_2SO_4>95% 46 ml) with stirring. A lot of heat will be released in this step, so an ice bath is used to keep the temperature below 20°C. Then the temperature was increased to 35°C and it was kept steady for 20 hours. This step was the main oxidization process. The π-structures in the carbon break down and the oxygen-containing functional groups become attached to the carbon backbone. Then 92 ml of pure water was added and kept at 98°C for 15 minutes. The suspension was further diluted with 280 ml pure water and 5 ml of hydrogen peroxide (H_2O_2) to reduce the residual permanganate and manganese dioxide concentration. Then the solid mixture was washed with UHQ to get rid of excess acid. The remaining materials were graphite oxide and normal graphite powder. Then a sonication process was applied to isolate the monolayer graphene oxide sheets from oxidized graphite and this resulted in a graphene oxide suspension with some impurities. Centrifugation at 8000 rpm was used to remove any solid residues after the sonication. Finally, a dialysis process (MW cut-off 14K) was applied for two weeks to get rid of the soluble impurities. This step was stopped when the conductivity of dialysis bath was below 2 µS for 24 hours.

S2. Speed and duration of spin-coating

A three step process of spin coating was used to create the surfaces for qSTORM experiments. Step 1 was used to deposit the GO film, step 2 for the polymer spacer and step 3 for the upper fluorophore coating. The spin coater parameters are shown in **Table S1**.

Step	Speed	Duration
1	300 rpm	10s
2	1000 rpm	15s
3	3000 rpm	45s

Table S1. Parameters used with the spin coater to create GO/polymer/fluorophore multi-layers for qSTORM experiments.

S3. Optical model parameters for ellipsometry fitting

The thicknesses of the polymeric layers (PMMA/polystyrene) were determined using a spectroscopic ellipsometer. A Cauchy model was used for the **graphene oxide** fit with the equation

$$\mathrm{n}(\lambda) = A_0 + \sum_{i>1} \frac{A_i}{\lambda^{2i}}$$

where n is the refractive index, λ is the wavelength, A_0=1.80, $A_i(i>0)=0$ and $k=\varepsilon=0$.

A Cauchy model was used for the **PMMA** fit with parameters

$A_0 = 1.480$ $A_1 = 0.006$ $A_i(i>1) = 0$ and $k = \varepsilon = 0$

A superposition of three oscillators model was used for the **polystyrene** fit.

1. Gaussian oscillator with position (E_n) = 4.920, amplitude (A_g) = 0.066960 and broadening (B_G) = 0.7755
2. Gaussian oscillator with position (E_n) = 5.674, amplitude (A_g) = 0.932530 and broadening (B_G) = 0.2736
3. Tauc-Lorentz oscillator with position (E_n) = 6.102, amplitude (A_g) = 167.6875, broadening (B_G) = 0.819 and band gap (E_G)=5.319

S4. Recipes of Imaging buffers

Antioxidant buffers were required to improve the longevity of the fluorophores in the qSTORM experiments, since photobleaching is closely related to oxidation and the uncertainty in measuring the position of the fluorophores scales with $\sqrt{N}$, where N is the number of photons emitted before bleaching.

Gloxy buffer:

50 mM β-MercaptoEthylamine hydrochloride (MEA),
10% (w/v) of glucose,
0.5 mg/ml glucose oxidase,
40 mg/ml catalase,
10mM NaCl,
in 40mM tris buffer pH adjusted to 8-8.5.

OxEA buffer:
50 mM β-MercaptoEthylamine hydrochloride (MEA),
3% (v/v) OxyFlour,
20% (v/v) of sodium DL-lactate solution,
in PBS, pH adjusted to 8–8.5.

S5. ThunderSTORM software used to construct super-resolution images

The ThunderSTORM plugin package (version 1.3 2014-11-08) for ImageJ was used to re-construct super-resolution images from each series of diffraction limited images[2]. The specific settings used for the software are shown below to help with reproducibility of the results (an optimal use of the software is important both for STORM imaging experiments and to calculate the energy transfers):

Image filtering
Filter function: Wavelet filter (B-Spline)
B-Spline order: 3
B-Spline scale: 2.0

Approximate localization of molecules
Method: Local maximum
Peak intensity threshold: 2×std(Wave.F1)
Connectivity: 8-neighbourhood

Sub-pixel localization of molecules
Method: PSF: Integrated Gaussian
Fitting radius [px]: 3
Fitting method: Weighted Least squares
Initial sigma [px]: 1.1
Multi-emitter fitting analysis: not enable

Visualisation of the results
Method: Scatter plot
Magnification: 10
Update frequency [frames]: 200
3D: not enable

S6. Localisations for the GO monolayers (figure 3)

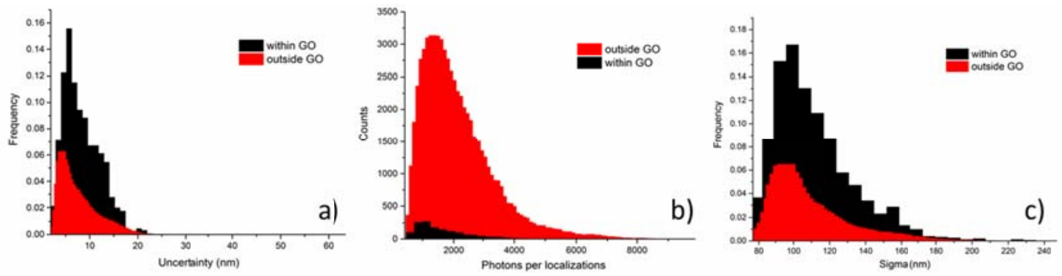

Figure S1. Histograms of a) the uncertainty, b) photons per localization and c) sigma within/outside GO area of **figure 3**. The mean value for each parameter is listed below.

Parameters	Within GO	Outside GO
Uncertainty (nm)	9	8
Photon per localization	1784	2236
Sigma (nm)	116	111

S7. Calculation of the energy transfer efficiency relation with the gap distance

The ratio of the total observed photons with a fluorophore at a distance r (Φ_r) from the graphene surface compared to at an infinite distance (Φ_∞)[3],

$$\frac{\Phi_r}{\Phi_\infty} = (1+\frac{\gamma_{nr}}{\gamma_r})^{-1} \qquad (1)$$

where γ_{nr} and γ_r are the non-radiative decay rate and the radiative decay rate respectively. The pure graphene (i.e. un-doped graphene) has little effect on the radiative decay of the fluorophore, so the decay rate ratio can be written as

$$\frac{\gamma_{nr}}{\gamma_r} \approx \frac{\gamma_{nr}}{\gamma_0} = \beta_1\gamma_1 + \beta_2\gamma_2 + \beta_3\gamma_3 \qquad \text{(e2)}$$

where β_i is the geometric factor, γ_0 is the decay rate in vacuum, and γ_i $(i = 1,2,3)$ are

$$\gamma_1 = \frac{9}{512\pi^3}\alpha\left(\frac{\lambda}{r}\right)^4 \qquad \text{(e3a)},$$

$$\gamma_2 = \frac{3}{64\pi}\alpha\left(\frac{\lambda}{2}\right)^2 \qquad \text{(e3b)},$$

$$\gamma_3 = \frac{3}{4}\pi\alpha[ci(ar)\cos(ar) + si(ar)\sin(ar)] \quad \text{and} \quad a = \frac{2\pi^2\alpha}{\lambda} \qquad \text{(e3c)},$$

where λ is the wavelength of the light emitted from the fluorophore, r is the distance between the graphene sheet and the fluorophore, α is the fine structure constant, *ci* and *si* are

standard sine and cosine integral functions respectively. In equation (e2) the first term on the RHS represents longitudinal coupling between the fluorophore and the graphene and it has an inverse fourth power dependence with the distance. The final two RHS terms come from the charged excitation and the second of these gives the quantitative asymptotic behaviour at large distances. Since the hexagonal lattice also exists in graphene oxide sheets, the model can also be used to describe the quenching effect of graphene oxide. The first RHS term in equation (e2) is the dominant one, so the energy transfer efficiency (ε) was fitted with the following form

$$\varepsilon = 1 - \frac{I_{GO}}{I_{NGO}} = 1 - [1 + A(\frac{\lambda}{z})^4]^{-1} \qquad \text{(e4)}$$

S8. Re-constructed images of a graphene oxide sheet with PMMA spacer layers and BSA conjugated to Alexa Fluor 647

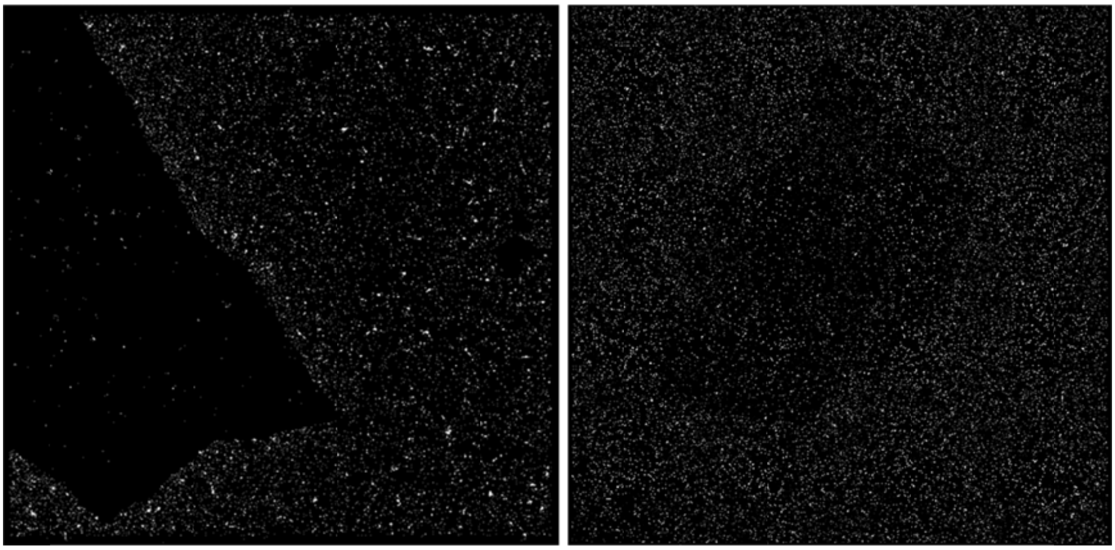

Figure S2. qSTORM images of single layer graphene oxide sheets using a coating of BSA conjugated to Alexa Fluor 647 with a PMMA spacer layer. The PMMA spacer thickness was 1 nm (left) and 10 nm (right).

Figure S2 shows super-resolution STORM images reconstructed from GO sheets with 1 nm and 10 nm thicknesses of PMMA spacer. BSA conjugated to the fluorophore Alex Fluor 647 was used to construct the images (as opposed to unconjugated CY3B). The Cy3B used in the main manuscript (**Figure 3**) has the advantage compared to the conjugated BSA that it is smaller and adsorbs flat to the surfaces, whereas BSA is more bulky, ellipsoidal and could adopt a variety of surface adsorbed conformations[4]. This tends to add to the uncertainties in contrast determination using BSA and equation 1.

S9. Fourier ring correlation plot

Fourier ring correlation plots were calculated for the self-assembled I_3K peptide super-resolved images[5]. On **figure S3** the left plot is for the sample without graphene oxide and the right plot is for the sample within the graphene oxide covered area. The FRC resolution calculated from **figure S3** improves slightly with the inclusion of GO i.e. it goes from 23 nm to 19 nm.

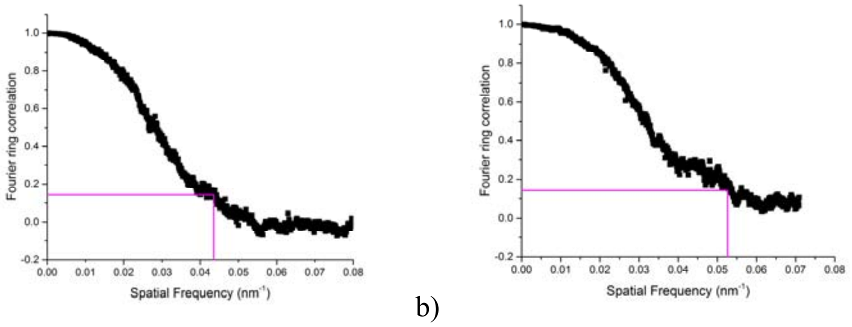

a) b)

Figure S3. Fourier ring correlation coefficients (FRC) as a function of spatial frequency for self-assembled I3K peptides a) without GO and b) with GO. The resolution calculated from these plots is 19 nm with GO and 23 nm without GO. The purple line shows the position of the threshold position at 1/7 and the corresponding spatial frequency used to determine the resolution.

Fourier ring correlation plots for the *E. coli* capsule images were also calculated. On **figure S4** the left plot is for the sample with graphene oxide and the right plot is for the sample without graphene oxide area. The resolution on **figure S4** improves slightly with the inclusion of GO i.e. it goes from 58 nm to 55 nm.

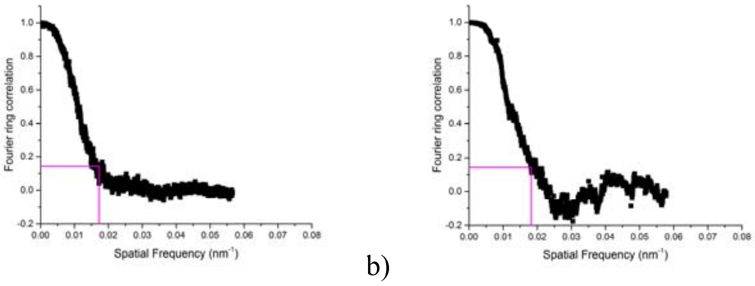

a) b)

Figure S4. Fourier ring correlation coefficient as a function of spatial frequency for *E. coli* capsule images a) with and b) without GO. The resolution calculated from these plots is 55 nm with GO and 58 nm without GO. The purple line shows the position of the threshold at 1/7 and the corresponding spatial frequency used to determine the resolution.

S10. Uncertainty of localisations

Localisation uncertainties for individual fluorophores were calculated using ThunderSTORM and are shown on **figure S5**. The left plot is the uncertainty distribution for the peptide sample and the right plot is for the *E. coli* sample. The red color indicates when there is no graphene oxide, whereas the black color is for the sample with a graphene oxide layer.

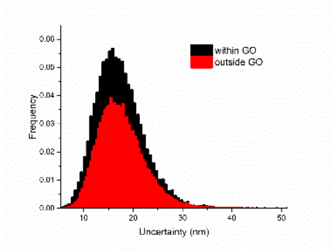

a)

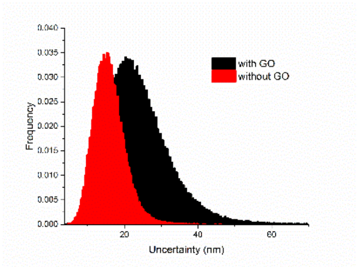

b)

Figure S5. Localization uncertainties for individual fluorophores calculated using ThunderSTORM. a) I3K peptides and b) *E. coli* capsules. The average uncertainties were a) 18 nm for peptides both with and without GO and b) 18 nm for *E. coli* without GO and 24 nm with GO.

The localisation uncertainty plays an important role in determining the image resolution in STORM experiments (**figures S3** and **S4**), but it is not the only determining factor[6]. We expect that the slight increase in localization uncertainty with *E. coli* and GO (**Figure S5b**) is due to a large population of bright fluorophores in the background that are extinguished by RET. However, this increased uncertainty does not significantly affect the image resolution in this case (**Figure S4**).

S11. The use of Fourier Ring Correlation to calculate the resolution of images with clumped fluorophore aggregates

Our GO coatings can effectively remove the signal from non-specifically bound dyes and other auto fluorescent debris and hence improve the signal to noise ratio. However, the Fourier Ring Correlation method is most sensitive to the localisation uncertainty and the number of localizations. By using GO covered substrates the photon number for each localization decreases due to the quenching effect. This leads to an increase in the average uncertainty for each localization fit. This is clearly shown in **figures S1** and **S5**. But the FRC resolution is not very sensitive to the signal to noise ratio (the contrast). In **figure S6** we provide a simulation to demonstrate this effect. The random grouped noise is included to simulate the clumped fluorophore artefacts that are common in fluorescence microscopy images and are observed in our experiments. The resolutions from Fourier Ring Correlation of super-resolved images c and d are 62 nm and 73 nm respectively. The large amount of clumped fluorophore artefacts in b and d strongly obscure the real signals, but they only have a small influence on the resolution calculated from FRC (about 18% reduced). A good FRC resolution implies details from all signals, both real sample and clumped noise artefacts, are well resolved.

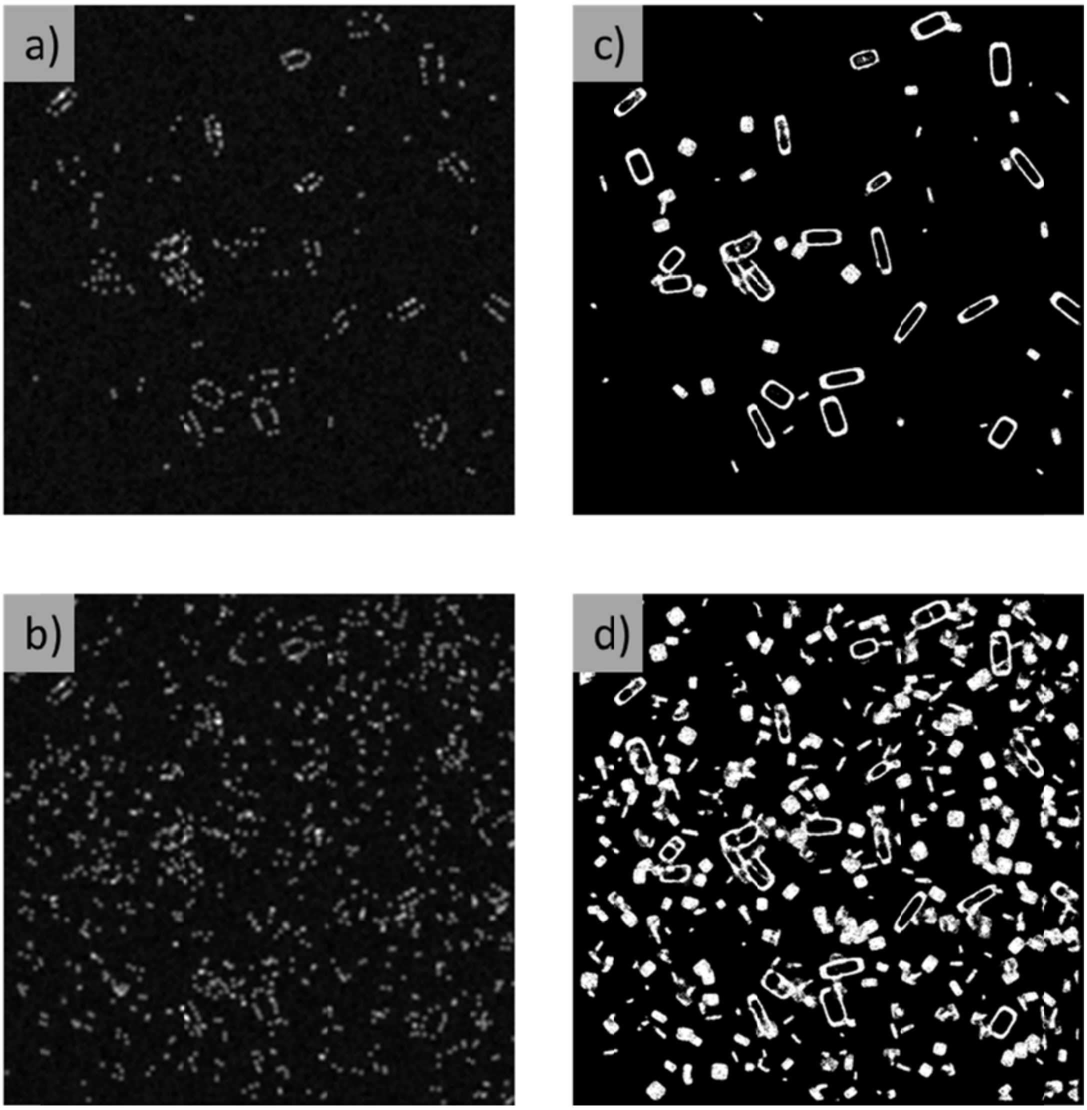

Figure S6. a) and b) are simulated diffraction limited images of 25 bacteria (elliptical shapes) with 50 and 550 regions of random grouped noise (irregular shapes) respectively. c) and d) are the corresponding scatter plot super-resolved images created from 1000 frames of the diffraction limited images (simulated in Matlab). The resolutions from Fourier Ring Correlation of super-resolved images c) and d) are 62 nm and 73 nm respectively. The large amount of clumped fluorophore artefacts in b) and) strongly obscure the real signals, but they only have a small influence on the resolution calculated from FRC (about 18% reduced). A good FRC resolution implies details from all signals, both real sample and clumped noise artefacts, are well resolved.

S12. Feature of interest metric for the image resolution

A feature of interest metric was introduced to overcome some of the issues experienced by the FRC metric in **S11**. It was demonstrated with peptide images, where the fibre thickness (11 nm from AFM) is conveniently below that of the effective point spread function (PSF) of the STORM technique i.e. cross sections through the fibres can be used to calculate the PSF. The peptide fibres were segmented using Fibreapp[7] and cross-sections were calculated through the fibres perpendicular to their contours using Gaussian fits. A representative Gaussian fit taken at a point along a fibre is shown in **figure S7**. A histogram of such Gaussians is shown in **figure 8** of the main manuscript.

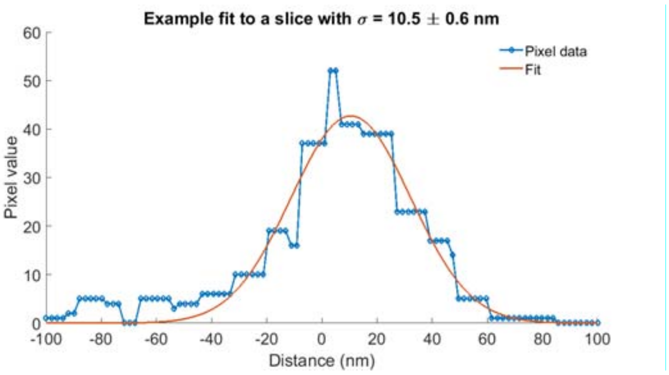

Figure S7. Example Gaussian fit through a segmented super-resolution image of a peptide fibre. The FWHM distributions are shown in **figure 8** of the main manuscript.

References

1 Hummers, W. S. & Offeman, R. W. Preparation of graphitic oxide. *Journal of American Chemical Society* **80**, 1339, doi:10.1021/ja01539a017 (1958).

2 Ovesny, M., Krizek, P., Borkovec, J., Svindrych, Z. & Hagen, G. M. ThunderSTORM: a comprehensive ImageJ plug-in for PALM and STORM data analysis and super-resolution imaging. *Bioinformatics* **30**, 2389-2390, doi:10.1093/bioinformatics/btu202 (2014).

3 Gomez-Santos, G. & Stauber, T. Fluorescence quenching in graphene: a fundamental ruler and evidence for tranverse plasmons. *Physical Review B* **84**, 165438, doi:10.1103/PhysRevB.84.165438 (2011).

4 Cowsill, B. J., Waigh, T. A., Eapen, S., Davies, R. & Lu, J. R. Interfacial structure and history dependent activity of immobilised antibodies in model pregnancy tests. *Soft Matter* **8**, 9847-9854, doi:10.1039/C2SM26133B (2012).

5 Nieuwenhuizen, R. P. J. *et al.* Measuring image resolution in optical nanoscopy. *Nature Methods* **10**, 557, doi:10.1038/nmeth.2448 (2013).

6 Mukammel, E. A. & Schnitzer, M. J. Unified resolution bounds for conventional and stochastic localization fluorescence microscopy. *Physical Review Letters* **109**, 168102, doi:10.1103/PhysRevLett.109.168102 (2012).

7 Usov, I. & Mezzenga, R. FiberApp: an open-source software for tracking and analyzing polymers, filaments and fibrous objects *Macromolecules* **48**, 1269-1280, doi:10.1021/ma502264c (2015).
